# Impact of physical self-perception in surgical result of patients with adolescent idiopathic scoliosis

**DOI:** 10.1186/1748-7161-7-S1-O10

**Published:** 2012-01-27

**Authors:** JF Sánchez-Soler, M Ramírez Valencia, J Martínez, J Bagó, L Andreu, L Puig

**Affiliations:** 1 Parc de Salut Mar - Barcelona, Spain; 2 Hospital Vall D'Hebron - Barcelona, Spain

## Background

A possible alteration of the self-image in patients with adolescent idiopathic scoliosis may influence the surgical result.

## Objective

Assess the correlation between the decrease in BMI and physical alteration of perception as measured by the Body Shape Questionnarie (BSQ-14) [1] in AIS with the end result perceived by patients after surgery.

## Materials and methods

Prospective study 32 patients surgery AIS from 2003 to 2006. 3 males-29 females. Mean age 15.9 years. BMI and Cobb angle was measured before surgery. After we measure Cobb, the physical alteration of perception by BSQ-14, the postoperative satisfaction with a questionnaire of 8 questions and the SRS-22 [2].Patients were grouped according to BMI, BMI <18 (group 1) and> 18 (group 2) and to BSQ-14 >40 (group 1a) and BSQ< 40 (group 2a).

## Results

The mean BMI was 19.6 kg /m, 37.5% a BMI <18. Mean preoperative Cobb 64.51° and the average correction rate of 56.57%.

Patients in group 1 showed worse results in the questions refered to the satisfaction of physical appearance after surgery. Group 1 also had poorer results of SRS-22 in self-image, subtotal dimension and the strong tendency in the dimension of satisfaction.

Patients in group 1a showed worse results the questions of satisfaction concerning self-image and overall satisfaction. In the SRS-22 in the dimension pain, self-image,satisfaction dimension, subtotal and total SRS.

We found no correlation between the degree curve correction with any questionnaire.

## Conclusions

Patients with a BMI <18 and patients with alteration of physical perception (BSQ> 40) have a worse surgical result.
